# Factors Associated with Visceral Fat Loss in Response to a Multifaceted Weight Loss Intervention

**DOI:** 10.4172/2165-7904.1000346

**Published:** 2017-08-14

**Authors:** FX Liu, SW Flatt, JF Nichols, B Pakiz, HS Barkai, DR Wing, DD Heath, CL Rock

**Affiliations:** 1 Department of Family and Preventive Medicine, School of Medicine, University of California, San Diego, 3855 Health Sciences Drive, La Jolla, CA, USA 92093-0901; 2 Exercise and Physical Activity Resource Center, University of California, San Diego, 3855 Health Sciences Drive, La Jolla, CA, USA 92093-0188

**Keywords:** Visceral fat, Diet, Portion-controlled foods, Physical activity

## Abstract

**Background:**

Visceral adipose tissue is more metabolically active than other fat depots and is more closely associated with obesity-related diseases, such as cardiovascular disease and type 2 diabetes, than indicators of obesity, such as body mass index. Across various strategies to promote weight loss, including energy-reduced diet and exercise, variable effects on VAT compared to loss of total body fat have been reported.

**Methods:**

To examine the effect of a behavioral weight loss intervention using portion-controlled prepackaged entrées on VAT, we examined data and measurements from overweight/obese men and women (N=183) who were assigned to a weight loss intervention and prescribed a reduced-energy diet with either portion-controlled prepackaged entrées or self-selected meals in a randomized clinical trial. VAT was estimated with dual-energy X-ray absorptiometry at baseline and study end (12 weeks).

**Results:**

VAT loss was greater for the prepackaged entrees group (p=0.02), with an average loss of 29% compared to an average loss of 19% among participants consuming self-selected meals. VAT (mean [SEM]) was 1651 (71) g and 1546 (157) g at baseline and 1234 (59) g and 1278 (118) g at study end in the prepackaged entrees and self-selected meal groups, respectively. Greater VAT loss was associated with higher baseline weight and VAT, and greater weight loss, but not associated with age or physical activity.

**Conclusion:**

Prescribing portion-controlled prepackaged entrees in a behavioral weight loss intervention promotes a reduction in VAT, which should promote improved metabolic profile and reduced cardiovascular disease risk.

## Introduction

Achieving and maintaining a healthy body weight has been a significant, yet difficult, goal for many people as evidenced by the high prevalence of obesity in the U.S. and other developed countries, and the plethora of weight loss strategies that exist [1]. However, it has also become clear that not all adipose tissue, and thus weight loss, is equal in importance for promoting health and reducing risk of chronic disease. For instance, android rather than gynoid distribution of fat is more often associated with metabolic syndrome, diabetes, and cardiovascular disease [2–7]. In particular, visceral adipose tissue (VAT), found mainly in the mesentery and omentum, has been described to be more metabolically active, more sensitive to lipolysis, and more insulin-resistant compared to subcutaneous fat. Further, VAT is more cellular, vascular, and innervated, and it contains a larger number of inflammatory and immune cells as compared to subcutaneous fat. Also, VAT has a greater capacity than subcutaneous fat to generate free fatty acids and to take up glucose [8–14].

Excessive VAT accumulation is associated with decreased production of adiponectin, an adipose-tissue derived molecule with metabolic and anti-inflammatory functions [15]. Increased VAT accumulation above a certain threshold is associated with decreased insulin sensitivity and increased cardiovascular disease risk in both males and females independent of total body fat [16]. Further, VAT is considered to be more closely associated with obesity-related diseases, such as cardiovascular disease and type 2 diabetes, than other indicators of obesity, such as body mass index (BMI), waist circumference, or waist-to-height ratio [17]. VAT volume was also found to be an independent predictor of elevated blood pressure, myocardial infarction, and insulin resistance [18].

Given the recognized importance of VAT and the demonstrated validity of using dual-energy X-ray absorptiometry (DXA) to estimate VAT [17,19], it is feasible and important to examine whether weight loss interventions not only cause loss of total fat but specifically loss of VAT and to describe the extent of loss. It is well-established that a reduction in energy intake is the primary dietary factor that is necessary to promote weight and fat loss and maintenance, and portion control is a strategy that may play a key role in weight management [20, 21] as well as promoting a reduction in VAT. We recently investigated whether providing portion-controlled prepackaged lunch and dinner entrées, compared to a standard self-selected diet, would promote weight loss in the context of a multifaceted intervention involving reduced-energy diet prescription and behavioral counseling [22]. We found that the meal plan incorporating portion-controlled prepackaged entrées promoted greater weight and fat loss than a standard self-selected diet, with comparable meal satisfaction [22]. The first specific aim of the present study was to examine whether VAT was differentially reduced across the meal plan group assignment in association with total weight and fat loss in the participants in that trial. A second specific aim was to examine the characteristics and factors associated with loss of VAT in this study population, because study participants were also encouraged to increase moderate and strenuous physical activity, which has been observed to independently reduce VAT [23].

## Materials and Methods

### Study population

As previously described [22], a multi-ethnic cohort of 77 men and 106 women with BMI 27-40 kg/m^2^ were enrolled into the study. The flow of participants during screening and study participation has been reported previously [22]. Age ranged from 25 to 65, with a mean of 46 years (Table 1).

The primary aim of the trial was to test whether providing portion-controlled prepackaged lunch and dinner entrées, compared to a standard self-selected diet, in the context of a reduced-energy diet prescription and behavioral counseling, promotes greater weight loss at 12 weeks in overweight and obese men and women. Participants were randomized 3:3:2 to receive prepackaged entrees, prepackaged higher protein entrees, or to select and prepare their own entrées and meals. As an exploratory study, half of those participants assigned to receive prepackaged entrees were provided only with items of a higher protein level (>25% energy), but the study’s statistical power was based on detecting a difference between the aggregated prepackaged food arms compared to the self-selected diet arm. Further, minimal difference in outcomes was observed between the two prepackaged entree groups so we analyzed the aggregated prepackaged entree group together for the current analysis.

### Intervention

As previously reported, all participants received personalized counseling to achieve an energy deficit sufficient to lose at least 5% of initial weight by three months, and they were encouraged to participate in at least 60 minutes of moderate intensity physical activity each day. Each participant met with a dietitian for an initial 1-2 hour personalized counseling session during which time they were prescribed an energy-reduced diet to obtain a deficit of 500-1500 kcal/day based on their individual weight loss goal. Use of Web-based and smart phone applications to monitor and guide diet and exercise recommendations were encouraged, and all participants were instructed to monitor energy intake and physical activity.

Participants assigned to the prepackaged entree group were asked to select from 50 varieties of frozen entrées (Lean Cuisine, Nestle USA, Inc., Glendale, CA, USA), or if assigned to the higher protein group, from the 25 entrées that provided >25% of energy from protein, to be consumed 7 days/week. Participants assigned to the self-selected diet group were provided a similar reduced-energy dietary prescription but were instructed to self-select all foods for their meals from the food groups and energy intake level prescribed. The meal plans for both groups aimed to achieve the myplate.gov nutrient composition and macronutrient distribution (45-65% energy from carbohydrate, 20-35% energy from fat, and 10-35% energy from protein).

### Measurements

Study data were collected and managed using a REDcap database hosted at the University of California, San Diego [24]. Participants completed clinic visits at baseline and 12 weeks during which weight, waist circumference, height (baseline only), and blood pressure were measured. Several questionnaires were also completed during these visits as well as collection of fasting (>6 hours) blood samples. Body composition was measured using a GE/Lunar Prodigy Dual Energy X-Ray Absorptiometer (DXA) [17, 19]. The following variables were examined: total body weight, total fat mass, percent fat, total android and gynoid fat, VAT, and lean mass. In a few subjects (n=5), visceral fat could not be estimated at baseline because the entire android region of the participant did not fit into the scan area.

Self-reported moderate and strenuous physical activity was assessed using the Godin Leisure-Time Exercise Questionnaire [25], a validated self-report measure of physical activity that has been widely used in previous research. We report weekly hours of moderate and strenuous physical activity.

### Statistical analysis

Longitudinal changes in the DXA measurements were compared within each diet group using paired t-tests. Changes between groups were compared using 2-sample t-tests. The percent of weight loss that was fat was also compared between the groups. We performed bivariate analysis to describe associations with change in VAT, change in % body fat, and change in lean mass, and then constructed three multivariate models to assess predictors of change in (VAT) change in % body fat and change in lean mass. Each model included group assignment, sex, age (binarized as older than the median age of 46 years, or not), baseline level of the outcome variable, baseline BMI, weight change at study end, strenuous physical activity reported at study end, and moderate physical activity reported at study end. We present the beta coefficients and standard errors of the mean for each factor potentially associated with the outcome measure. All analysis was done with SAS version 9.4 (SAS Institute, Cary, NC).

## Results

Along with significant decreases in body weight, there were concurrent significant reductions in both study groups in total and percent body fat; android fat, gynoid fat, VAT, and lean body mass (Table 2).

Subjects in the pre-portioned prepackaged entree group lost more weight and more total fat than those in the self-selected diet as previously reported [22], and in this analysis, we also found larger decreases in android fat, VAT, and lean body mass (Table 3) Total weight loss and loss of total fat, VAT, and lean body mass were significantly correlated (data not shown), as would be expected.

The multivariate model for change in VAT (Table 4) shows that VAT loss was associated with greater overall weight loss, baseline BMI, and baseline VAT. Those whose baseline BMI was above the sample median (>33 kg/m^2^) lost a mean of 466 (44) g of VAT and 1.8 (0.2) kg of lean mass compared with 332 (35) g of VAT fat and 1.6 (0.2) kg of lean mass in those whose baseline BMI was below 33 kg/m^2^. Participants with less than 1600 g of VAT at baseline lost an average of 219 g of VAT, compared with 587 g of VAT among subjects with >1600 g of VAT at baseline. Also, those who achieved a 5% weight loss lost a mean of 483 g of VAT and 2.2 kg of lean mass compared with 156 g of VAT and 0.3 kg of lean mass in those who did not achieve a 5% weight loss. Likewise, change in total percent fat was associated with baseline BMI and overall weight change. Although strenuous physical activity was not associated with change in VAT, it was associated with change in total body percent fat and with change in lean mass. Higher amounts of strenuous physical activity were associated with smaller decreases in lean mass. Subjects who engaged in at least 2.6 hours of strenuous physical activity weekly lost 1.6 (0.2) kg of lean mass, and their body fat decreased by 3.4 percentage points (from 41.0 to 37.6%). Subjects with lower amounts of strenuous activity lost 1.8 (0.2) kg of lean mass, and their body fat decreased by 2.5 percentage points (from 41.5 to 39.0%). Men lost an average of 2.4 kg of lean mass, and women lost an average of 1.2 kg. Absolute change in lean mass was inversely associated with baseline lean mass. Age and moderate physical activity were not significantly associated with changes in VAT, total percent fat or lean body mass.

**Figure 1:**
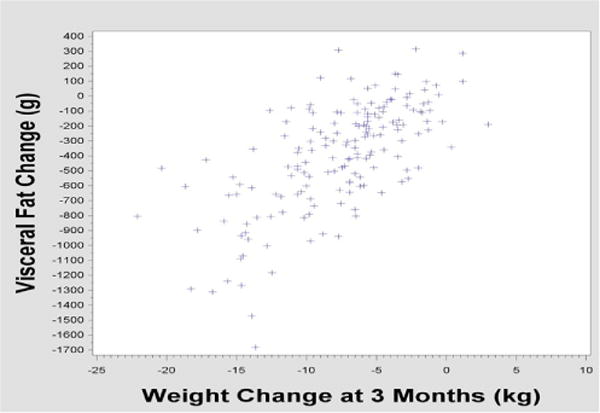
Plot of visceral fat g change (y axis) vs weight loss in kg (x axis).

We saw no evidence of a threshold effect below which VAT loss was insignificant; change in VAT loss appeared to correlate linearly with overall weight loss (Figure 1).

## Discussion

A reduced-energy intake facilitated by prescribing portion-controlled prepackaged entrées, in addition to increased physical activity, resulted in greater loss of VAT, as well as total fat and percent fat over the 3-month intensive behavioral weight loss intervention. Notably, participants in both study groups (pre-portioned prepackaged entrées and self-selected meals) lost a significant percentage of VAT with the prepackaged entrées group losing more. There was an average loss of 29% VAT among participants receiving-portion controlled meals, compared to an average loss of 19% among participants consuming self-selected meals. Also, we found that having a higher baseline level of VAT, a higher baseline BMI, and a greater amount of weight loss over the study period was associated with a greater loss of VAT. This represents a large change over a short time period and is important given the metabolic significance of VAT compared to subcutaneous and other body fat, which can lead to significant improvement in overall health [7–9,10]. Lean mass also decreased significantly in association with greater weight loss, but this is an expected and well-documented outcome of intentional weight loss efforts, although not necessarily desirable [26,27].

The mechanism by which this intervention may have promoted VAT loss most likely is the combined effects of energy restriction and aerobic exercise. Other studies have shown that both reduced energy diets and aerobic exercise in particular have can dramatic effects on VAT reduction [23,28–30]. One study found that there was a dose– response relationship between aerobic exercise and VAT reduction in subjects without metabolic-related disorders [29]. Results of that study also indicated that the degree of VAT loss can be directly attributed to the amount of aerobic exercise. In the present study, we did not find a significant independent link between physical activity, strenuous or otherwise, and the degree of VAT loss. This could be due to limitations in the self-reported nature of the measure of physical activity or that the effect from energy restriction greatly overwhelmed the effect of exercise in this particular study.

Our findings are consistent with several other studies that suggest VAT can be significantly reduced during a weight loss intervention [28,30,31] and a similar correlation between baseline level and loss has been previously reported. Previous studies have observed that VAT is preferentially lost in the early part of a weight loss intervention due to increased sensitivity of VAT lipid metabolism-related genes to fasting and preferential sympathetic nervous system regulation of VAT depots [28,32,33]. Given that the weight loss intervention was three months in length, our observation was in a relatively early stage of weight loss. Further weight loss has been suggested to attenuate or even terminate further VAT loss [28]. In the present study, we identified an association between VAT loss and total weight loss, and we did not find a threshold below which VAT was lost to a greater or lesser extent. Notably, body composition measures in this study were only performed at the beginning and end of the three-month study period, so incremental VAT loss is not definable in greater detail over the study time frame.

Age and sex are also possible factors associated with VAT accumulation and loss, with visceral fat reported to increase with age and increase more in males than females [34,35]. In the present study, we did not find age or sex to be independently associated with loss of VAT. Age and sex may have had an indirect effect in our study where older male participants with more baseline VAT also had a greater loss of VAT not because of age or sex but simply because of their higher baseline VAT, as would be characteristic of their demographic characteristic [34, 35]. Because small subgroups would have substantially constrained statistical power, differences in responses across race/ethnicity categories was not examined in this study, but it has been suggested that there can be variation in VAT loss based on race [31].

There are several strengths of this study, including the near equal distribution of women and men, the large proportion of participants from minority racial/ethnic groups, and measurement of body composition by DXA. Also, the low drop-out rate minimizes ambiguity in drawing inferences from this study. A limitation of the study includes the lack of detailed dietary intake data. Participants were encouraged to self-monitor dietary intake through various tools and technology of their choice, but standardized dietary recalls or records were not conducted or collected. The sample was a free-living population, so variability in adherence is likely. Self-reported data for physical activity have well-recognized limitations in accuracy, characterized as over reporting and misreporting especially among individuals who are overweight or obese. Further, DXA scans were obtained only at the beginning and end of the study which did not allow examination of the timeline of VAT loss during the three months of the weight loss intervention.

## Conclusion

In conclusion, prescribing portion-controlled prepackaged entrées in the context of an intensive behavioral weight loss intervention not only promotes loss of total fat but also a significant reduction in VAT, which can promote an improved metabolic and lipid profile and also reduce cardiovascular disease risk.

## Figures and Tables

**Table 1:** Participant characteristics at study enrollment.

Characterstics	Prepackaged entrees (n = 138)	Self-selected meals (n = 45)
Age, yrs, mean	46.2 (0.9)	45.6 (1.6)
Sex %		
Female	55.8	64.4
Male	44.2	35.8
Weight, kg, mean	95.9 (1.3)	95.9 (2.3)
BMI, kg/m^2^, mean	33.1 (0.3)	33.5 (0.6)
Moderate/strenuous activity hours/week, mean	2.6 (0.2)	2.2 (0.3)

**Table 2:** Longitudinal changes in body composition.

Variable	Prepackaged entrées (n =138) Mean	Self-selected meals (n = 45) Mean
Total fat, kg		
Baseline	39.0 (0.7)	40.3 (1.3)
3 months	33.2 (0.8)	36.1 (1.2)
p value	<0.0001	<0.0001
% Fat		
Baseline	41.1 (0.6)	42.3 (1.0)
3 months	37.8 (0.7)	40.2 (1.0)
p value	<0.0001	<0.0001
Android fat, kg		
Baseline	3.9 (0.1)	3.9 (0.2)
3 months	3.1 (0.1)	3.4 (0.2)
p value	<0.0001	<0.0001
Gynoid fat, kg		
Baseline	6.0 (0.1)	6.5 (0.2)
3 months	5.1 (0.1)	5.7 (0.2)
p value	<0.0001	<0.0001
Visceral fat, g		
Baseline	1651 (71)	1546 (137)
3 months	1234 (59)	1278 (118)
p value	<0.0001	<0.0001
Lean mass, kg		
Baseline	53.5 (1.0)	52.3 (1.7)
3 months	51.7 (1.7)	51.2 (1.7)
p value	<0.0001	0.001
Weight, kg		
Baseline	95.9 (1.3)	95.9 (2.3)
3 months	87.8 (1.3)	90.1 (2.2)
p value	<0.0001	<0.0001

a Analysis used paired t-tests, comparing baseline and 3 months within group.

**Table 3:** Changes in body composition between pre-portioned and self-selected meal groups.

Variable	Prepackaged entrees (n = 138) Mean	Self-selected meals (n = 45) Mean	P value^a^
Total fat, kg			
Change at study end	−5.7 (0.3)	−4.4 (0.5)	0.03
% Fat			
Change at study end	−3.1 (0.2)	−2.4 (0.3)	0.06
Android fat, kg			
Change at study end	−0.8 (0.1)	−0.5 (0.1)	0.01
Gynoid fat, kg			
Change at study end	−0.9 (0.1)	−0.8 (0.1)	0.17
Visceral fat, g			
Change at study end	−0.4 (0.03)	−0.3 (0.04)	0.02
Lean mass, kg			
Change at study end	−1.9 (0.2)	−0.9 (0.2)	0.001
Weight, kg			
Change at study end	−7.9 (0.4)	−5.8 (0.7)	0.01
% Loss that was fat	65.4 (3.7)	70.5 (3.5)	0.32

a Two-sample t-test for difference in change scores between prepackaged entrées and self-selected meal groups at study end.

**Table 4:** Results of multivariate analysis.

Variables	Change in visceral fat (g) (R^2^ = 0.68)s	–	Change in % total fat (R^2^ = 0.59)	–	Change in lean mass (g) (R^2^ = 0.51)	–
Factor	Beta	P value	Beta	P value	Beta	P value
Prepackaged entrées	−14.21 (39.55)	0.72	0.326 (0.277)	0.24	−559.0 (240.1)	0.02
Male sex	−33.05 (43.52)	0.45	−0.618 (0.422)	0.15	788.2 (397.8)	0.05
Baseline level of outcome variable	−0.225 (0.029)	<0.0001	−0.061 (0.033)	0.07	−0.062 (0.018)	0.001
3-Month strenuous physical activity	−5.78 (5.75)	0.32	−0.106 (0.040)	0.01	78.73 (35.11)	0.02
3-Month moderate physical activity	−2.78 (6.50)	0.67	−0.060 (0.046)	0.2	76.88 (39.77)	0.06
Age below median (< 46 yrs)	−6.38 (36.48)	0.86	−0.214 (0.235)	0.36	60.55 (204.8)	0.77
Baseline body mass index	20.55 (5.54)	0.0003	0.188 (0.044)	<0.0001		0.008
Weight change at 3 months	41.94 (4.18)	<0.0001	0.360 (0.029)	<0.0001		<0.0001

## Abbreviations

BMI: Body mass index
DXA: Dual energy X-ray absorptiometry
VAT: Visceral adipose tissue
